# Supplementary material for: The Role of Vitamin D Supplementation in Enhancing Muscle Strength Post-Surgery: A Systemic Review
Source: Nutrients. 2025 Apr 29;17(9):1512. doi: 10.3390/nu17091512 (PMC12073290; doi:10.3390/nu17091512)
Supplement: Supplementary file 1 [file nutrients-17-01512-s001.zip › Supplemenatry File S1.pdf]

S1: Complete Search Strategy Utilised

Pubmed

Search	Results
(Vitamin D[MeSH] OR Ergocalciferols[MeSH] OR Ergosterol[MeSH] OR Cholecalciferol[MeSH] OR Vitamin D Deficiency[MeSH]) OR (Vitamin D*[Title/Abstract] OR Vit D*[Title/Abstract] OR Ergocalciferol[Title/Abstract] OR Cholecalciferol[Title/Abstract] OR Calcitriol[Title/Abstract] OR "25-hydroxyvitamin D*"[Title/Abstract] OR "Sunshine Vitamin*"[Title/Abstract] OR "D-hormone*"[Title/Abstract])	126,334
(Surgery[MeSH] OR General Surgery[MeSH]) OR (postoperative*[Title/Abstract] OR post-operative*[Title/Abstract] OR Postsurgical*[Title/Abstract] OR post-surgical*[Title/Abstract] OR surger*[Title/Abstract] OR surgical[Title/Abstract] OR "Surgical follow-up outcome*"[Title/Abstract] OR "Post-surgical prognosis"[Title/Abstract] OR "Surgical aftereffect*"[Title/Abstract] OR "Recovery outcomes after surgery"[Title/Abstract])	4,992,919
(Muscle Strength[MeSH]) OR ("Muscle Strength"[Title/Abstract] OR "Muscle function"[Title/Abstract] OR "Muscular strength"[Title/Abstract] OR "Muscle strength"[Title/Abstract] OR "Muscle fitness"[Title/Abstract] OR "Muscle performance"[Title/Abstract] OR "Musculoskeletal health"[Title/Abstract] OR "Muscle endurance"[Title/Abstract] OR "Muscle vitality"[Title/Abstract] OR "Skeletal muscle health"[Title/Abstract] OR "Muscle resilience"[Title/Abstract] OR "Muscle capacity"[Title/Abstract])	88,039
#1 AND #2 AND #3	**92**

Embase

Search	Results

vitamin D/ or vitamin D deficiency/ or Vitamin D.mp. or Ergocalciferols.mp. or ergocalciferol/ or Ergosterol.mp. or ergosterol/ or Cholecalciferol.mp. or colecalciferol/ or (Vitamin D* or Vit D* or Ergocalciferol or Cholecalciferol or Calcitriol or 25-hydroxyvitamin D* or Sunshine Vitamin* or D-hormone*).ab,kf,ti.	208005
surgery/ or surgery.mp. or general surgery/ or (postoperative* or post-operative* or postsurgical* or post-surgical* or surger* or surgical or Surgical follow-up outcome* or Post-surgical prognosis or Surgical aftereffect* or Recovery outcomes after surgery).ab,kf,ti.	5217205
Muscle Strength.mp. or muscle strength/ or (Muscle Strength or Muscle function or Muscular strength or Muscle fitness or Muscle performance or Musculoskeletal health or Muscle endurance or Muscle vitality or Skeletal muscle health or Muscle resilience or Muscle capacity).ab,kf,ti.	125645
#1 AND #2 AND #3	**262**

Cochrane

Search	Results
(Vitamin D[MeSH] OR Ergocalciferols[MeSH] OR Ergosterol[MeSH] OR Cholecalciferol[MeSH] OR Vitamin D Deficiency[MeSH]) OR (Vitamin D*[Title/Abstract] OR Vit D*[Title/Abstract] OR Ergocalciferol[Title/Abstract] OR Cholecalciferol[Title/Abstract] OR Calcitriol[Title/Abstract] OR "25-hydroxyvitamin D*"[Title/Abstract] OR "Sunshine Vitamin*"[Title/Abstract] OR "D-hormone*"[Title/Abstract])	40104
(Surgery[MeSH] OR General Surgery[MeSH]) OR (postoperative*[Title/Abstract] OR post-operative*[Title/Abstract] OR Postsurgical*[Title/Abstract] OR post-surgical*[Title/Abstract] OR surger*[Title/Abstract] OR surgical[Title/Abstract] OR "Surgical follow-up outcome*"[Title/Abstract] OR "Post-surgical prognosis"[Title/Abstract] OR "Surgical aftereffect*"[Title/Abstract] OR "Recovery outcomes after surgery"[Title/Abstract])	407547

(Muscle Strength[MeSH]) OR ("Muscle Strength"[Title/Abstract] OR "Muscle function"[Title/Abstract] OR "Muscular strength"[Title/Abstract] OR "Muscle strength"[Title/Abstract] OR "Muscle fitness"[Title/Abstract] OR "Muscle performance"[Title/Abstract] OR "Musculoskeletal health"[Title/Abstract] OR "Muscle endurance"[Title/Abstract] OR "Muscle vitality"[Title/Abstract] OR "Skeletal muscle health"[Title/Abstract] OR "Muscle resilience"[Title/Abstract] OR "Muscle capacity"[Title/Abstract])	69183
#1 AND #2 AND #3	**348**
